# Glucolipid Metabolic Disorders and *Helicobacter pylori* Infection

**DOI:** 10.1155/ije/8479537

**Published:** 2026-02-10

**Authors:** Jing Yuan, Pingjie Xiong, Zhipeng Zhou, Jiali Wu, Baihua Wu, Bin Wang, Jiao Guo

**Affiliations:** ^1^ Guangdong Metabolic Diseases Research Center of Integrated Chinese and Western Medicine, Guangdong Pharmaceutical University, Guangzhou, China, gdpu.edu.cn; ^2^ Key Laboratory of Glucolipid Metabolic Disorder, Guangdong Pharmaceutical University, Ministry of Education, Guangzhou, China, meb.gov.tr

**Keywords:** glucolipid metabolic disorders, *H. pylori*, mechanism

## Abstract

Due to its high incidence and severe consequences, glucolipid metabolic disorders (GLMD) remain a significant challenge for the global medical community. *Helicobacter pylori* (*H. pylori*), a Gram‐negative bacterium that colonizes the gastric mucosa, has a high infection rate worldwide, exceeding 50% in the global population. Although numerous studies have explored the associations between *H. pylori* and individual metabolic diseases, a systematic review framework that integrates these findings to provide a comprehensive perspective on GLMD is still lacking. Recent studies have indicated that *H. pylori* infection can lead to disturbances in lipid and glucose metabolism; however, this area of research is fraught with controversy, even yielding completely opposing conclusions. Based on this, we systematically reviewed the research on the association between *H. pylori* and type 2 diabetes (T2DM), metabolic dysfunction–associated steatotic liver disease (MASLD), and atherosclerosis (AS). We conducted an in‐depth analysis of how *H. pylori* infection influences the onset and progression of GLMD through multiple mechanisms, including the induction of inflammatory responses, exacerbation of oxidative stress, and impairment of insulin sensitivity. Simultaneously, we highlighted the roles of bacterial virulence factors and the exosomes they regulate in metabolism. In conclusion, we have determined that *H. pylori* infection may induce inflammatory responses, exacerbate oxidative stress, and impair insulin sensitivity by regulating the levels of inflammatory cytokines, adiponectin (ADPN), leptin, vitamin D, homocysteine, and exosomes, thereby collectively influencing the occurrence and progression of the aforementioned diseases. Therefore, effective control and treatment of *H. pylori* infection should not be neglected in the management of GLMD.

## 1. Introduction

GLMD are characterized by disturbances in glucose and lipid metabolism caused by various factors, including genetics, environment, psychology, and diet. The core pathological mechanisms primarily involve neuroendocrine dysregulation, insulin resistance, oxidative stress, chronic inflammation, and gut microbiota imbalance. The main clinical manifestations include hyperglycemia, dyslipidemia, fatty liver, and AS [1]. GLMD have become a significant public health issue that severely threatens human health worldwide. Controlling risk factors such as blood lipids and blood glucose is key to their prevention and treatment. Therefore, in‐depth research into the influencing factors of glucose‐lipid metabolism is of great significance for the prevention and control of GLMD. In addition to common factors such as genetics, diet, and environment, it is also essential to explore and study other factors that affect lipid and glucose metabolism. *H. pylori*, a Gram‐negative bacterium that colonizes the gastric mucosa, has a high infection rate globally, exceeding 50% in the natural population [2]. Numerous studies have indicated that *H. pylori* infection can lead to various metabolic disturbances, particularly in lipid and glucose metabolism. However, research findings vary significantly across different countries and populations, with some studies even reaching completely opposite conclusions. A recent bidirectional Mendelian randomization study indicated no genetic evidence of a causal relationship between *H. pylori* and MASLD, suggesting that the eradication or prevention of *H. pylori* infection may not benefit MASLD [3]. In a study conducted by Seoul National University in 2011 involving 462 elderly participants with an average age of 66.2 years, it was found that after adjusting for confounding factors, individuals infected with *H. pylori* had higher low‐density lipoprotein cholesterol (LDL‐C) levels than those who were uninfected, with an odds ratio of 3.1, suggesting that *H. pylori* promotes AS by altering lipid levels [4]. Conversely, many opposing viewpoints suggest that there is no correlation between *H. pylori* infection and elevated lipid levels, and that blood lipid levels did not improve after *H. pylori* eradication. For instance, studies conducted in Japan from 2002 to 2004 found that the number of individuals with hyperlipidemia and obesity actually increased after the eradication of *H. pylori* [5–7]. Another study involving 124 patients found that the eradication of *H. pylori* was associated with a long‐term decrease in hemoglobin A1c levels, particularly in male subjects and those younger than 65 years [8]. In 2012, Naja et al. conducted a study on insulin resistance and metabolic syndrome in adults, revealing that *H. pylori* infection was not associated with the incidence of T2DM or insulin resistance [9].

Given the considerable controversy surrounding the relationship between *H. pylori* infection and glucose‐lipid metabolism in various studies, this research systematically summarizes and analyzes the association between and T2DM, MASLD, and AS. It was found that *H. pylori* infection may influence the development of GLMD by affecting the levels of cytokines such as tumor necrosis factor‐α (TNF‐α), interleukin‐1β (IL‐1β), interleukin‐17 (IL‐17), interleukin‐6 (IL‐6), ADPN, and leptin, as well as vitamin D, homocysteine, and exosomes.

## 2. The Relationship Between *H. pylori* and T2DM

### 2.1. Evidence of the Association Between *H. pylori* Infection and T2DM

In 1989, Simon et al. first published a research report on the relationship between *H. pylori* infection and diabetes [10]. Subsequently, in a case‐control study, we found that *H. pylori* infection is not only a risk factor for diabetes but also has a positive correlation with diabetes [11, 12], and this relationship is significant in women [13]. In fact, Jeon et al. found in a prospective cohort study that included 782 Latin American elderly participants that *H. pylori*‐positive patients had a 2.7 times increased risk of developing diabetes compared to those who were *H. pylori*‐negative [14].

Relatedly, Aydemir et al. first confirmed the association between chronic *H. pylori* infection and insulin resistance through a homeostasis model assessment [15]. They found that the insulin resistance index in *H. pylori*‐positive patients was significantly higher than that in the *H. pylori*‐negative group. Ludovico Abenavoli and colleagues observed that after treating insulin‐resistant patients with *H. pylori* eradication therapy, the insulin resistance index returned to normal levels [16]. In a cohort study, researchers found that long‐term *H. pylori* infection increases levels of glycated hemoglobin, insulin resistance, and triglycerides (TG) [17]. After eradicating *H. pylori*, levels of glycated hemoglobin and insulin resistance decrease [8].

### 2.2. Cytokines

The cytokines play an important regulatory role in the initiation and progression of T2DM induced by *H. pylori* infection, such as TNF‐α, IL‐1β, IL‐6, IL‐17, and interferon‐gamma (IFN‐γ). Specifically, *H. pylori* regulates the phosphorylation of p65 at the Ser‐536 site through integrin‐linked kinase, thereby activating the nuclear factor kappa B(NF‐κB) signaling pathway and promoting the secretion of TNF‐α [18]. Both TNF‐α and IL‐1β have been shown to affect insulin sensitivity and induce the occurrence of T2DM by modulating the secretion of insulin by pancreatic β‐cells.

In patients infected with *H. pylori*, the expression levels of IL‐17 and IL‐6 are significantly higher [19, 20]. A meta‐analysis found that IL‐6‐mediated inflammation is associated with T2DM [21]. Increased expression of SOCS‐3 induced by IL‐6 affects insulin signaling [22]. In addition, The heat shock protein 60 derived from *H. pylori* may stimulate T Helper 1 Cell lymphocytes to secrete IFN‐γ through cross‐reactivity with human heat shock protein 65 antibodies, resulting in reduced insulin secretion [23–25].

Individuals infected with *H. pylori* typically exhibit high levels of leptin and low levels of ADPN. It has been observed that after eradicating *H. pylori*, leptin levels decrease while adiponectin levels increase [26, 27]. A systematic review found that the levels of ADPN significantly decrease in patients with T2DM [28] (Table 1).

**Table 1:** The mediating roles of key factors in *H. pylori* infection linking to T2DM, MASLD, and AS (this original table was created by the authors to illustrate).

Key factors	T2DM	MASLD	AS
TNF‐α	1. Stimulate SOCS3, inhibit the degradation of IRS‐1 and IRS‐2 proteins [29, 30]. 2. TNF‐α and IL‐1β cause insufficient secretion of pancreatic β‐cells through the L‐arginine‐dependent nitric oxide synthesis pathway [31, 32];	1. It also inhibits the expression and activity of CYP7A1, which decreases the hepatic breakdown of total cholesterol, causing excessive accumulation of TG in hepatocytes [33]. 2. Stimulates the production of TGF‐β, thereby activating the differentiation and proliferation of hepatic stellate cells [34]. 3. TNF‐α induces periostin through cJun, which subsequently promotes collagen deposition [35].	1. TNF‐α can inhibit the production of NO in endothelial cells [36]. 2. TNF‐α and IL‐1β promote the retention of LDL in the vascular wall by increasing their transcytosis, thereby facilitating AS [37, 38].
IL‐1β		IL‐1β can promote apoptosis of hepatocytes and lead to liver fibrosis by activating macrophages and neutrophils [39].	IL‐1β induces smooth muscle cell proliferation and migration via the JAK2/STAT3 signaling pathway [40].
IFN‐γ	IFN‐γ will activate the tryptophan/kynurenine pathway, leading to islet cell dysfunction [41].	/	1. IFN‐γ, through the activation of the JAK and STAT signaling cascades, elicits oxidative burst, which in turn fosters the accumulation of foam cells [42]. 2. It stimulates the proliferation of smooth muscle cells and disrupts plaque stability [42]. 3. IFN‐γ can upregulate indoleamine 2,3‐dioxygenase, leading to a decrease in serum tryptophan levels and an increase in kynurenine metabolite levels [43]. 4. Generates ROS, leading to endothelial damage [44].
IL‐17	IL‐17 can initially activate NF‐κB, further activate the JNK pathway, and induce neutrophil infiltration into the islets, leading to a decrease in insulin sensitivity and β‐cell function [45].	1. IL‐17 stimulates Kupffer cells to express IL‐6, IL‐1β, TNF‐α, and TGF‐β1 [46], and activates hepatic stellate cells to produce type I collagen, promoting liver fibrosis [35, 47, 48]. 2. IL‐17 inhibits fatty acid β‐oxidation, promotes TG accumulation [49], and accelerates hepatic steatosi	IL‐17 can stimulate the differentiation of macrophages and the secretion of pro‐inflammatory cytokines, as well as increase the expression of TLR [50].
IL‐6	1. The C‐174G polymorphism in the promoter region of the IL‐6 gene has been shown to affect insulin sensitivity [51]. 2. IL‐6 activates SOCS‐3, which inhibits the tyrosine phosphorylation of insulin receptor substrates, thereby affecting insulin signaling [52–54].	1. Significantly reduces IRS‐1‐related PI 3 kinase activity, affecting insulin signaling and glucose uptake. 2. Increased tyrosine phosphorylation of STAT3 3. Reduce the level of fatty acyl‐CoA in the liver. 4. Promotes SOCS‐3‐mediated degradation of IRS‐1 and IRS‐2, leading to defects in insulin signaling and action [55, 56].	1. Increase the levels of foam cells formed after the binding of Ox‐LDL cholesterol, leading to the accumulation of lipids within macrophages [57]. 2. Induces endothelial dysfunction, activating and sustaining vascular inflammation [58].
Leptin	Inhibit insulin secretion and production by beta cells [59].	1. Increased expression of TNF‐α in the liver promotes the development of NASH [60]. 2. Increased expression of transforming growth factor beta 1 leads to the activation of hepatic stellate cells [61]. 3. Increasing the levels of ROS in the liver prompts Kupffer cells to produce TNF‐α and other cytokines, which promote collagen production and hepatic fibrosis [62].	1. Leptin induces chronic oxidative burst in endothelial cells [63]. 2. Stimulates the expression of thrombospondin‐1 through JAK2/STAT and MAPK signaling pathways, as well as the activation of ERK1/2 and NF‐kB, or affects the migration and proliferation of vascular smooth muscle cells by enhancing MMP‐9 expression [64–69]. 3. It can impair ERK1/2 and eNOS signaling in endothelial cells by upregulating the expression of caveolin‐1 protein, leading to endothelial vascular dysfunction [70]. 4. Increase the expression of cell adhesion molecules, thereby damaging endothelial cells [71, 72].
ADPN	Reducing glucose utilization and insulin sensitivity by inhibiting AMPK activity [73].	1. Stimulating the proliferation of hepatic stellate cells by reducing AMPK activity [74]. 2. Inhibiting AMPK, p38 MAPK, and PPAR‐α to regulate liver fatty acid metabolism [75].	1. Low levels of ADPN not only stimulate lipopolysaccharide‐induced TNF‐α production [76] 2. Upregulation of macrophage scavenger receptor MSR promotes the transformation of macrophages into foam cells [77].
Vitamin D	Vitamin D stimulates the secretion of insulin by pancreatic beta cells [78].	1. Reduce AMPK phosphorylation levels, increase IL‐1β and caspase‐1 activity, and enhance lipid peroxidation [79]. 2. Inhibit M1 macrophage polarization to reduce lipid accumulation in hepatocytes [80].	Elevating JNKp promotes PPARγ expression, which facilitates foam cell formation [81].
Exosome	1. The miR‐155 present in exosomes can inhibit the expression of PPAR‐γ, leading to reduced insulin signaling [82]. 2. M1‐like macrophage‐derived exosomes impair β‐cell insulin secretion through miR‐212‐5p by targeting SIRT2 and inhibiting the protein kinase b/GSK‐3β/β‐catenin pathway [83]	1. Elevating levels of β‐catenin, α‐SMA, and TIMP1 proteins, while downregulating E‐cadherin, promotes hepatic stellate cell activation [84]. 2. Extracellular vesicles regulate hepatic stellate cells by targeting microRNAs that activate peroxisome proliferator‐activated receptor gamma [85].	1. Reducing the expression of transcription factors PPARγ and LXRα inhibits the transcription of cholesterol efflux transport proteins, thereby exacerbating foam cell formation [86]. 2. In endothelial cells, the activation of the NF‐κB signaling pathway and pro‐inflammatory transcription factor STAT3 accelerates the inflammatory process [87]. 3. Activated ROS/NF‐κB, thereby promoting endothelial cell proliferation [88].

Secondly, extracellular vesicles in *H. pylori*‐infected cells are categorized into three subtypes: exosomes, microvesicles, and apoptotic bodies. Exosomes serve as regulators of intercellular and interorgan crosstalk in metabolism, comprising a large number of proteins, lipids, microRNAs, mRNAs, and noncoding RNA species. *H. pylori* can stimulate gastric epithelial cells to secrete exosomes, inducing an increase in exosomes [89], which can reduce insulin sensitivity and the secretion of insulin from β cells (Table 1).

Finally, vitamin D levels in patients with *H. pylori* infection tend to be lower [90]. Research on obese subjects with T2DM has found that vitamin D deficiency may be associated with the onset of T2DM [91], as VDR and vitamin D‐dependent calcium‐binding proteins are present in beta cells [92], which stimulate insulin secretion by beta cells. Therefore, low levels of vitamin D may lead to insulin resistance and impaired beta‐cell function, further promoting the development of T2DM [93] (Table 1).

## 3. *H. pylori* and MASLD

### 3.1. Evidence of the Association Between *H. pylori* Infection and MASLD

Last year, nonalcoholic fatty liver disease (NAFLD) was renamed for the second time as MASLD [94, 95]. The latest studies indicate that persistent infection with *H. pylori* increases the risk of MASLD and may promote liver fibrosis and liver damage [96, 97]. A recent meta‐analysis has disclosed a notable and direct association between *H. pylori* infection and the presence of moderate to severe NAFLD, reinforcing the link between these two conditions [98]. Among diabetic individuals, *H. pylori* infection emerges as a risk enhancer for NAFLD development. Effective management of blood glucose levels and the eradication of *H. pylori* infection could potentially yield beneficial effects in reducing the prevalence of NAFLD in this patient population [99]. Additionally, cytotoxin‐associated gene A (CagA)and vacuolating cytotoxin A (VacA) are important virulence factors of *H. pylori*, and the seroprevalence of CagA and VacA was found to be significantly associated with MASLD [100].

### 3.2. Cytokines

*H. pylori* infection affects glucose metabolism, lipid metabolism, and inflammatory factors in patients with MASLD [101]. Contemporary studies have illuminated that *H. pylori* elicits inflammatory reactions by perturbing the intricate immune microenvironment within the gastric mucosa. This disruption encompasses the complex interplay between *H. pylori*, host immune cells, and diverse immune modulators, underscoring its role in instigating inflammatory processes [102]. These proinflammatory cytokines contribute to the progression of NAFLD by engaging diverse inflammatory cascades, ultimately leading to the disruption of insulin signaling pathways [103, 104]. There is a study indicating that *H. pylori* is capable of inhibiting neutrophil apoptosis and exacerbating inflammation by inducing IL‐1β secretion through autophagy [105].

In fact, *H. pylori* can also activate the c‐Jun N‐terminal kinase (JNK) inflammation signaling pathway through toll‐like receptor 6 [106]. The activation of the JNK inflammation signaling pathway is closely related to liver damage [107]. In addition, *H. pylori* downregulates miR‐203 by affecting the gene expression transcription factor c‐Jun, which increases the level of the SOCS3 insulin suppressor. This leads to a reduction in insulin signaling and ultimately results in hepatic insulin resistance [108].

In the context of NAFLD, *H. pylori* infection has been shown to stimulate toll‐like receptor 4 within liver tissue via the p38 MAPK pathway. This activation triggers macrophages, particularly through P38*α*, to secrete TNF‐α and IL‐1β, and promote M1 polarization [109]. Research has found that *H. pylori* activates NF‐κB signaling through the virulence factor CagA, leading to an increased expression of interleukin‐17A [110]. TNF‐α and interleukin‐17A lead to excessive accumulation of TG in the liver (Table 1). Moreover, the excessive accrual of TG within the liver can precipitate hepatic insulin resistance by bolstering gluconeogenesis and activating pivotal kinases such as protein kinase C‐ε and JNK1, further exacerbating metabolic dysregulation [111]. Studies have already demonstrated that TNF‐α and IL‐17 promote liver injury and liver fibrosis [112]. Furthermore, IL‐1β is involved in the development of liver fibrosis (Table 1).

ADPN and leptin also play important roles in the relationship between *H. pylori* and NAFLD [113, 114]. In large prospective NAFLD studies, elevated serum leptin levels have been observed [115]. High levels of leptin are closely associated with the development and progression of liver inflammation and fibrosis [116] (Table 1). A recent study has shown that the ADPN concentration in NAFLD patients is lower than in normal patients [117]. AdipoR2 can increase the levels of free fatty acids in the cytoplasm, thereby promoting hepatocyte damage. The increase in free fatty acids production activates activating the protein kinase C in the cytoplasm through the synthesis of fatty acid derivatives, leading to the phosphorylation of Insulin Receptor Substrate‐1, which contributes to insulin resistance and promotes lipid deposition in the liver, resulting in hepatocyte damage [118].

### 3.3. Deficiency of Vitamin B12 and Vitamin D

Long‐term *H. pylori* infection may elevate homocysteine levels [119], negatively impacting the absorption and metabolism of folic acid and vitamin B12 [120]. Vitamin B12 and folic acid are essential cofactors in the metabolism of methionine. Deficiencies in these factors can lead to methylation failure and hyperhomocysteinemia, which may lead to hyperhomocysteinemia. *H. pylori* infection induces hyperhomocysteinemia, which can activate the Nod‐like Receptorprotein3 inflammasome through ubiquitination of heat shock factor 1, leading to insulin resistance, hepatic steatosis, and further development of NAFLD [121, 122]. Moreover, research has found that individuals with *H. pylori* positivity exhibit reduced levels of vitamin D compared to those without the infection [90, 123]. *H. pylori* may promote NAFLD by affecting vitamin D levels (Table 1).

## 4. *H. pylori* and AS

### 4.1. Evidence of the Association Between *H. pylori* Infection and AS

The latest research indicates that *H. pylori* infection contributes to the development of AS [124]. A cross‐sectional study found a significant correlation between chronic inflammation induced by *H. pylori* and AS [125]. In a cohort study of 2626 adults, it was found that *H. pylori* infection significantly affects lipid levels [126].

The risk of dyslipidemia was lower after eradication of *H. pylori*, suggesting that eradication can improve HDL levels and reduce the risk of dyslipidemia [126, 127]. In addition, myocardial infarction is the most severe manifestation of AS. According to a meta‐analysis, the infection rate of *H. pylori* in patients with acute myocardial infarction is 2.1 times higher than that in the control group [128]. In conclusion, current research findings indicate that *H. pylori* infection is closely associated with AS.

### 4.2. CagA and VacA

In recent years, numerous studies have revealed the role of *H. pylori* bacterial outer vesicles [103, 129, 130]. CagA and VacA are two important virulence factors expressed by *H. pylori*, and they play a crucial role in the pathogenesis of this bacterium.

VacA is an exotoxin secreted by *H. pylori*. Recent studies have found that after being internalized by gastric epithelial cells, VacA localizes to the inner mitochondrial membrane, forming ion‐conducting channels that lead to mitochondrial dysfunction. This dysfunction impairs the host cell’s metabolism through ATP production, increasing the oxidation and deposition of LDL, which promotes the occurrence of AS [131].

CagA is a virulence factor protein expressed by *H. pylori*. When infected with *H. pylori*, the host’s immune system recognizes the CagA protein and produces antibodies against it. Antibodies against CagA can cross‐react with antigen proteins related to vascular smooth muscle cells and endothelial cells, leading to damage and dysfunction of vascular endothelial cells, which in turn contributes to the occurrence and progression of AS [132]. Furthermore, CagA disrupts the pro‐inflammatory transcription factor STAT3 in endothelial cells, accelerating AS [86, 87].

Research shows *H. pylori*‐infected cells release CagA‐exosomes into blood, causing extra‐gastric diseases [133]. On one hand, exosomal CagA can induce the release of proinflammatory mediators [134], and on the other hand, these exosomes derived from gastric epithelial cells infected with *H. pylori* regulate the expression of ABCA1, ABCG1, and SR‐BI by downregulating the PPARγ‐LXRα signaling pathway, leading to cholesterol accumulation in macrophages and accelerating the formation of foam cells [86].

### 4.3. Cytokines

During *H. pylori* infection, macrophages are recruited to the gastric mucosa, where, under the influence of bromodomain‐containing protein4, they regulate glycolysis and polarize into the M1 phenotype [135]. M1 macrophages secrete a significant amount of Pro‐inflammatory cytokines, such as TNF‐α, IL‐1β, and IL‐6 [136–138]. Many studies have shown that IL‐6 is involved in the development of AS [139, 140]. IL‐6 maintains vascular inflammation by promoting smooth muscle cell proliferation and migration, endothelial dysfunction, and the recruitment and activation of inflammatory mediators, further leading to plaque instability [141]. In addition, *H. pylori* stimulates T Helper 1 cells to secrete IL‐17 and IFN‐γ. Among them, IFN‐γ not only promotes the accumulation of foam cells but also facilitates the occurrence of AS by reducing tryptophan levels (Table 1). Increasing evidence suggests that leptin contributes to AS by promoting endothelial cell damage and smooth muscle cell proliferation [64, 142, 143]. Jin Peng et al. believe that ADPN can protect cardiovascular health by improving lipid metabolism, protecting vascular endothelial cells, inhibiting foam cell formation, and suppressing the proliferation of vascular smooth muscle cells [144]. It may also contribute to the polarization of macrophages in peripheral fat tissue through STST6 signaling, thereby improving AS [145].

It is well known that when infected with *H. pylori*, CRP levels increase. CRP activates the complement system by binding in a calcium‐dependent manner to phosphatidylcholine on oxidized low‐density lipoprotein (Ox‐LDL), forming CRP complexes. This process facilitates the localization of CRP with the terminal membrane attack complex C5b‐9, which further induces the proliferation of smooth muscle cells and activates the NF‐κB signaling pathway, ultimately promoting the formation of foam cells [145–149].

Additionally, elevated levels of CRP may lead to the upregulation of DNA methyltransferase3B expression in response to Ox‐LDL. DNA methyltransferase3B mediates hypermethylation at multiple sites in the promoter region of the CREG (cellular repressor of E1A‐stimulated genes), resulting in decreased expression of CREG. This alteration lowers the levels of phosphorylated eNOS, leading to impaired endothelial relaxation function and is associated with CRP‐induced AS [150, 151]. On the other hand, matrix MMPs produced by macrophages can degrade collagen in the fibrous cap of atherosclerotic plaques, potentially resulting in plaque rupture [152], which may trigger myocardial infarction.

It is noteworthy that elevated fibrinogen and thrombin levels are associated with *H. pylori* infection and fibrinogen is also a known risk factor for AS [153, 154]. The level of fibrinogen significantly decreases after successful eradication of *H. pylori* [155]. Fibrinogen exhibits a dual role by binding to intercellular adhesion molecule‐1 present on endothelial cells and additionally upregulating the expression of adhesion molecules on these cells, thereby enhancing their adhesive capacity [156]. Moreover, it prompts the release of vasoactive factors, ultimately resulting in the augmentation of endothelial permeability [157]. In addition, fibrinogen deposits adsorb LDL‐C and lead to atherosclerotic plaque formation [158, 159].

Finally, *H. pylori* infection affects the secretion of gastrointestinal hormones, which can have significant impacts on cardiovascular health. Low levels of ADPN not only activate endothelial cells but also promote the occurrence and development of AS by facilitating the formation of foam cells and reducing the production of nitric oxide (Table 1).

### 4.4. Formation of Foam Cells and Platelet Aggregation

Macrophages occupy a pivotal position in mediating metabolic signaling processes and inflammatory responses within the context of atherosclerotic plaques, thereby exerting a fundamental influence on their development and progression [135]. They participate in plaque AS by absorbing Ox‐LDL and forming foam cells [160]. Under normal circumstances, macrophages absorb LDL particles through low‐density lipoprotein receptors. The expression of low‐density lipoprotein receptors is downregulated due to elevated cholesterol levels, which limits the absorption of LDL‐C and maintains normal levels.

**Figure 1:**
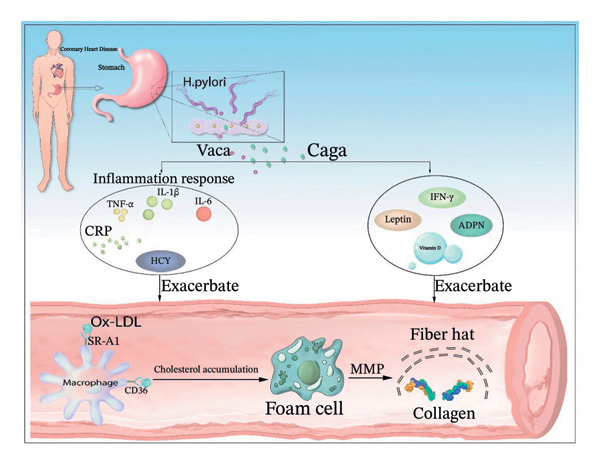
*H. pylori* infection exacerbates the formation of foam cells (this original figure was created by the authors to illustrate). After being transported to various tissues and organs, CagA and VacA secreted by *H. pylori* jointly promote the binding of scavenger receptors CD36 and SR‐A1 on macrophages to Ox‐LDL, leading to their conversion into cholesterol crystals. This process, along with the inflammatory responses induced by TNF‐α, IL‐1β, CRP, IL‐6, and Hcy, as well as changes in the levels of ADPN, leptin, IFN‐γ, and Vitamin D, results in the accumulation of cholesterol crystals and the formation of foam cells. Ultimately, under the action of MMP, the collagen in the fibrous cap is degraded. Note: *H. pylori*: *Helicobacter pylori*, TNF‐α: Tumor necrosis factor‐α, IL‐1β: Interleukin‐1β, IL‐6: Interleukin‐6, Hcy: Homocysteine, LDL: Low‐density lipoprotein, MMP: Matrix metalloproteinase, IFN‐γ: Interferon‐gamma, CagA, cytotoxin‐associated gene A, VacA: Vacuolating cytotoxin A, ADPN: Adiponectin, CRP: C‐reactive protein.

Nonetheless, *H. pylori* infection can lead to elevated levels of LDL‐C. LDL undergoes oxidative modification into Ox‐LDL through enzymes and ROS. The oxidized fatty acids and phospholipid constituents present in Ox‐LDL amplify the capacity of endothelial cells to upregulate ICAM‐1 and vascular cell adhesion molecule‐1, which subsequently facilitates the recruitment of monocytes and their transformation into macrophages [161–163]. At the same time, Ox‐LDL does not bind to low‐density lipoprotein receptors but instead interacts with scavenger receptors SR‐A1 and CD36. Because SR‐A1 and CD36 are not downregulated with cholesterol levels, cholesterol continues to accumulate, ultimately resulting in the uptake of oxidized LDL‐C by macrophages and the formation of foam cells (Figure 1).

On the other hand, since CD36 is a common ligand‐binding receptor for Ox‐LDL, the scavenger receptor CD36 binds to Ox‐LDL and transforms into cholesterol crystals, resulting in the accumulation of cholesterol crystals [164]. Subsequently, cholesterol crystal–mediated complement activation results in the upregulation of complement receptor 3 (CD11b/CD18), facilitating cholesterol crystals phagocytosis. This process triggers lysosomal damage, subsequently activating the Nod‐Like Receptorprotein3 inflammasome and caspase‐1, leading to the secretion of IL‐1β. Ultimately, this cascade culminates in cell death and of plaque the progression formation [165, 166], This further promotes the occurrence and development of AS. At the same time, the accumulation of lipoproteins facilitates the formation of L‐selectin‐ and P‐selectin‐dependent platelet‐leukocyte aggregates on leukocytes, which favors the secretion of chemokines by endothelial cells [167], such as platelet factor 4 and PAF. These factors induce aggregation by binding to PAF receptors [168]. This favors the recruitment of immune cells, thereby exacerbating the inflammatory response mediated by proinflammatory cytokines, further promoting AS.

In addition, *H. pylori*‐infected subjects had elevated serum von Willebrand factor. Initially, *H. pylori* strains bind von Willebrand factor and bind to glycoprotein Ib [169] on the surface of platelets, while *H. pylori* urea binds to phospholipase A2, promoting the release of adenosine diphosphate [170]. Adenosine diphosphate and thrombin produced by *H. pylori* together promote cell activation by stimulating receptors linked to heterotrimer G proteins, which induce platelet activation through different signals, and then bind integrin αIIbβ3 on the platelet surface to plasma fibrinogen to mediate platelet aggregation [171]. *H. pylori* infection also increased levels of cyclooxygenase‐1 and cyclooxygenase‐2 result in elevated levels of TXA2 and PGE2, which promote platelet aggregation [172].

### 4.5. Deficiency of Vitamin B12 and Vitamin D

*H. pylori* can affect vitamin D levels, and lower vitamin D levels increase the risk of developing AS [173]. Vitamin D is present in vascular smooth muscle [174] and cardiomyocytes [175], regulating their reproduction and growth. Low levels of vitamin D not only lead to reduced eNOS activity, significantly decreased levels of nitric oxide, and vascular dysfunction [176], but also result in the accumulation of lipids within macrophages. Foam cells deposit lipid streaks in the vascular endothelia, and these lipid streaks will gradually form plaques, leading to vascular aging and decreased elasticity.

Then, when *H. pylori* causes an increase in homocysteine concentration, it can form complex [177] with LDL, which is subsequently phagocytozed by macrophages to form foam cells, increasing the stiffness of the arteries leading to AS. The study found that homocysteine can induce the increase of CRP mRNA expression in smooth muscle cells through the NMDAr‐ROS‐MAPK‐NF‐κB signaling pathway, which leads to inflammatory response and further aggravates the formation of foam cells [178].

## 5. Conclusion

Numerous studies have demonstrated a significant association between *H. pylori* infection and the development of T2DM, MASLD, and AS. This review, building upon a systematic synthesis of existing evidence, further reveals that *H. pylori* infection may not merely induce isolated metabolic disorders through single‐factor alterations, but rather contribute to the concurrent progression of multiple interrelated metabolic diseases via multifaceted mechanisms. *H. pylori* infection is posited to modulate a broad “*H. pylori*–cellular messenger–metabolism” regulatory network involving TNF‐α, IL‐1β, IL‐17, IL‐6, ADPN, leptin, vitamin D, and exosomes, thereby influencing the common pathogenic pathways underlying GLMD (Table 1). Specifically, it drives the release of inflammatory cytokines via activation of key signaling pathways, generating an inflammatory cascade that induces insulin resistance and accelerates T2DM progression; promotes lipid accumulation, hepatic insulin resistance, and oxidative stress, thereby facilitating MASLD development; and induces inflammatory responses and oxidative stress that enhance macrophage uptake of Ox‐LDL, foam cell formation, and plaque progression, ultimately leading to AS. Consequently, this systematic analysis offers a novel perspective for clinical practice, suggesting that eradicating *H. pylori* in combination with anti‐inflammatory, antioxidant, and insulin‐sensitizing strategies may help reduce the prevalence of GLMD. Future research should focus on exosomal CagA and VacA levels, which may serve as potential biomarkers for identifying individuals at risk of developing GLMD following *H. pylori* infection, thereby enabling targeted interventions. In summary, this review not only establishes a multifactorial mechanistic framework linking *H. pylori* to GLMD but also highlights its potential role in GLMD pathogenesis. However, the exact causal relationship between *H. pylori* and GLMD remains contentious and warrants further investigation, particularly regarding the roles of bacterial virulence factors, exosomes, and vitamin metabolism.

## Author Contributions

Jing Yuan drafted the manuscript. Pingjie Xiong made critical revisions to important knowledge content. Zhipeng Zhou made critical revisions to important knowledge content. Jiali Wu made critical revisions to important knowledge content. Baihua Wu made critical revisions to important knowledge content. Bin Wang made critical revisions to important knowledge content. Jiao Guo designed this review.

## Funding

This work was supported by the National Key R&D Program of China (2023YFC3606200), the National Natural Science Foundation of China (T2341005), and the Major Basic and Applied Basic Research Projects in Guangdong Province of China (2019B030302005)

## Disclosure

All claims expressed in this article are solely those of the authors and do not necessarily represent those of their affiliated organizations, or those of the publisher, the editors, and the reviewers. Any product that may be evaluated in this article, or claim that may be made by its manufacturer, is not guaranteed or endorsed by the publisher. All authors participated in the final editing of the manuscript and approved the submitted version.

## Conflicts of Interest

The authors declare no conflicts of interest.

## Data Availability

Data sharing not applicable to this article as no datasets were generated or analyzed during the current study.
